# Subchronic administration of scopolamine reverses UCMS-induced behavior in mice via eEF2 protein dephosphorylation

**DOI:** 10.1007/s43440-024-00630-4

**Published:** 2024-07-23

**Authors:** Yana Babii, Agnieszka Pałucha-Poniewiera, Anna Rafało-Ulińska, Piotr Brański, Andrzej Pilc

**Affiliations:** Department of Neurobiology, Maj Institute of Pharmacology, Polish Academy of Sciences, 12 Smętna Street, Kraków, 31-343 Poland

**Keywords:** Scopolamine, Antidepressant, Depression, Chronic mild stress, eEF2

## Abstract

**Background:**

The cholinergic system has been increasingly linked to the pathophysiology of mood disorders such as depression, with the potential involvement of nicotinic and/or muscarinic receptors. Conventional antidepressants usually require weeks of daily dosing to achieve a full antidepressant response. In contrast, clinical studies have shown that scopolamine, a nonselective muscarinic acetylcholine receptor antagonist, can induce potent and rapid antidepressant effects, requiring only a few days of treatment. This study aimed to examine the suitability of the unpredictable chronic mild stress (UCMS) model of depression to reproduce the above scopolamine antidepressant activity patterns.

**Methods:**

Rapid and sustained antidepressant-like effects were assessed by using the splash test, sucrose preference test (SPT), tail suspension test (TST), and forced swimming test (FST) in animals undergoing the UCMS procedure and stress-naïve C57BL/6J mice. Western Blotting was used to measure tropomyosin receptor kinase B (TrkB), mammalian target of rapamycin (mTOR), eukaryotic elongation factor (eEF2) and postsynaptic density protein 95 (PSD95) levels.

**Results:**

Scopolamine induced antidepressant-like effects in a dose-dependent manner only after subchronic, but not single, administration in the UCMS model of depression in C57BL/6J mice without affecting locomotor activity. Specifically, scopolamine administered at a dose of 0.3 mg/kg for four consecutive days significantly reversed the UCMS-induced depressive-like behavior, such as apathy, anhedonia, and behavioral despair, while scopolamine, given at the same dose but only once, did not relieve the above symptoms. Scopolamine treatment was accompanied by eEF2 protein dephosphorylation and its subsequent reactivation in the prefrontal cortex (PFC).

**Conclusion:**

Subchronic administration of scopolamine is needed to ameliorate UCMS-induced depressive-like behavior. The suggested mechanism of scopolamine action covers eEF2 protein activity in the PFC.

**Supplementary Information:**

The online version contains supplementary material available at 10.1007/s43440-024-00630-4.

## Introduction

Depression is a chronic, severe, and recurrent disorder. Among all mental disorders, depression is the most common cause of partial incapacity in both professional and private life. Depression not only impairs health in its own right, but also increases the risk of other health outcomes, as well as suicide [1–3]. First introduced over 60 years ago, conventional antidepressants have revolutionized the treatment of mood disorders. However, currently accessible first-line antidepressants are known for their delayed onset of action, low response rate, and notable side effects, which hamper their use as an appropriate therapy for many patients [4]. Hence, there is a clinical need for alternative antidepressant drug targets being able to become a rapid-acting and effective therapeutic options. A new hope in depression treatment are hallucinogenic substances such as scopolamine belonging to a subclass of deliriant hallucinogens. Scopolamine is a nonselective acetylcholine muscarinic (AChM) receptor antagonist. Scopolamine has been used for years in the form of butylbromide (does not penetrate through the blood-brain barrier, BBB) as an antispasmodic agent, and in the form of hydrobromide (crosses BBB) for the treatment of motion sickness and postoperative nausea [5, 6]. Since the 1970s, research has revealed the involvement of cholinergic system alterations in the pathophysiology of mood disorders. Studies involving acetylcholinesterase inhibitors, such as physostigmine or donepezil, and cholinergic agonists like arecoline or oxotremorine, have indicated a potential association with depressed moods [7–10]. Notably, individuals exposed to insecticides or nerve agent weapons– also acetylcholinesterase inhibitors– have shown the development of psychiatric symptoms, including depression [11]. Nevertheless, it was not until 2006 that Furey and Drevets first described a relatively rapid and long-lasting antidepressant effect in patients with major depression, including those who did not respond to conventional antidepressants [12]. Numerous subsequent replications have further validated these findings across diverse treatment populations [13–16].

However, given that deliriants remain the least explored group of hallucinogens, their intricate and enigmatic nature requires additional preclinical and clinical studies. Even though scopolamine exerts rapid, but not single-dose induced, antidepressant effect, there is a notable gap in understanding its full potential. On the other hand, one of the best attempts to mirror the human state in animals is the unpredictable chronic mild stress (UCMS) model, which simulates uncontrolled stress experienced by individuals [17]. Therefore, our present study concerns the normalizing effects of scopolamine treatment, investigating how rapid and sustained its therapeutic effects are [18]. During the UCMS procedure, mice are exposed to a set of heterotypic physical and neurogenic stressors in an unpredictable manner, minimizing habituation– a decrease in the response magnitude to a specific stimulus due to its repeated exposure [19]. The initial dosages of scopolamine were determined based on preliminary experiments and literature values [20].

Furthermore, we investigated the scopolamine mechanism of action, particularly focusing on the possible role of tropomyosin receptor kinase B (TrkB), mammalian target of rapamycin (pmTOR/mTOR), eukaryotic elongation factor 2 (peEF2/eEF2) and PSD95 protein in observed effects.

## Materials and methods

### Animals and housing

C57BL/6J male mice, aged 6–7 weeks and weighing 23–25 g at the beginning of the experiment, were purchased from Charles River Laboratories (Germany) and were housed individually in cages with food and tap water provided ad libitum. The animals were kept under standard laboratory conditions of lighting (12-h light/dark cycle), humidity (55 ± 10%), and temperature (22 ± 2 °C). Each experimental group consisted of eight to ten animals. All experiments were performed in accordance with the guidelines of the National Institutes of Health Animal Care and Use Committee and were approved by the Second Local Ethics Committee in Krakow, Poland. All efforts were made to minimize the number of animals used and their suffering. Considering that behavioral diversity is partially sex-dependent, and since the primary objective of the experiment was not to compare male and female behavior, the study limited its focus to male mice only.

### Compounds

Scopolamine hydrobromide (Tocris Cookson Ltd., Bristol, UK) was dissolved in 0.9% NaCl. Groups of control animals were randomly chosen and received equal volumes of the vehicle alone (0.9% NaCl). Tested compounds were prepared immediately before administration and administered intraperitoneally (ip) at a constant volume of 10 ml/kg.

### Unpredictable chronic mild stress procedure

**Fig. 1:**
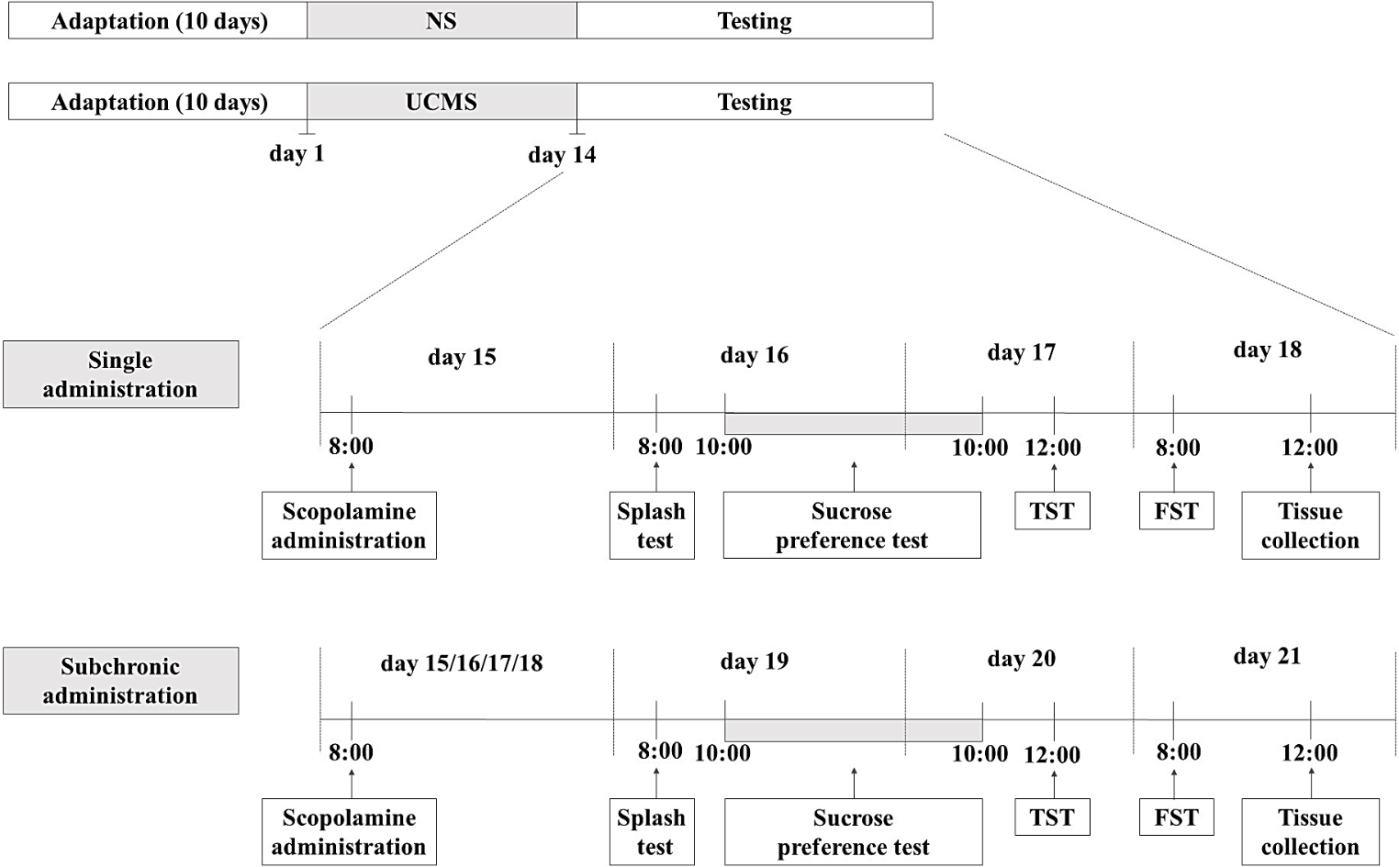
The schedule of the UCMS experiments. NS– non-stressed; UCMS– unpredictable chronic mild stress

UCMS model of depression was established in male C57BL/6J mice, following a previously outlined methodology [21]. The mice were randomly divided into two groups: one group remained unstressed (referred to as NS), while the other group underwent the UCMS procedure (referred to as UCMS). To eliminate potential stress from olfactory, visual, and auditory stimuli, these two groups were housed in separate rooms. After ten days of acclimation to the room conditions, the UCMS procedure was initiated, with stressors and their durations outlined in Table 1. Chronic sleep disruption was also considered as an additional stressor, given that stressors were applied during the light phase when rodents, being nocturnal animals, are typically asleep [22]. Two stressors were chosen daily, spanning 14 procedure days. Importantly, consecutive days did not involve the same stressor to ensure unpredictability, with a minimum gap of two hours between stressors. On the 15th day afterthe procedure initiation, the animals were treated with two concentrations of scopolamine (0.1 or 0.3 mg/kg) or a vehicle, administered either singly or subchronically (for four consecutive days). Then, 24 h after the last (single or subchronic) treatment, a set of behavioral tests, including the splash test, SPT, TST, and FST, was performed. Control mice underwent the same treatment and testing regimen as the UCMS mice. The detailed schedule of the UCMS procedures is illustrated in Fig. 1. Two hours after the last behavioral test, the animals were sacrificed by decapitation, and specific brain structures, namely the PFC and hippocampus, were collected and frozen at -80 °C.

**Table 1:** Stressors used in unpredictable chronic mild stress protocol

Stressor	Duration
restraint stress	1 h
cage tilting 45°	6 h
wet bedding	2 h
predator smell (rat)	2 h
removal of sawdust	1 h
placing a mouse in the cage of another mouse	2 h
two male mice in one cage	2 h
3–6 individuals in a cage with 37 °C water	30 min
overcrowding (12 individuals)	1 h

### Behavioral tests

All behavioral experiments were conducted in a dedicated testing room during the light period (8:30 − 14:30) of the light/dark cycle. After a 24-hour interval following the last treatment, three parameters reflecting the core symptoms of depression were analyzed. These parameters included reduced grooming time in the splash test, signifying apathy; diminished sucrose preference, indicative of anhedonia; and decreased periods of immobility observed in both the TST and FST tests, resembling behavioral despair. The order and time intervals between behavioral tests are based on our previous studies, considering the severity and likelihood of one test interfering with the results of another [21, 23]. This approach allows animals to fully recover from the previous test and ensures better reliability for subsequent tests. Additionally, locomotor activity was tested to assess any non-specific effects of the antidepressant drugs.

### Splash test

The splash test has been performed as previously described [24] with slight adjustments. The test was conducted under subdued lighting in a room where the animal was acclimated for 30 min. To induce self-grooming behavior, a thick 10% sucrose solution was sprayed onto the dorsal fur of the mice. The sprayer consistently dispensed the sucrose solution (approximately 0.2 ml), with each mouse receiving five sprays. Then, the grooming activity duration was monitored over five minutes.

### Sucrose preference test (SPT)

The SPT was conducted following the protocols established by Strekalova and Steinbusch [25] and Pałucha-Poniewiera et al. [24], with minor modifications. One day before the test, mice were given a palatable 2.5% sucrose solution for 2 h to reduce the impact of neophobia. Then, the actual SPT began, during which mice had unrestricted access to two bottles (1% sucrose solution and tap water) for 24 h, with the positions of the bottles switched after 12 h. There was no prior food or water deprivation before the test. At the start and end of the test, the bottles were weighed, and liquid consumption was measured. The sucrose preference rate was calculated using the following equation:

$$\eqalign{& Sucrose\;preference\;rate\;\left( \% \right) = \cr & \quad {\eqalign{& The\;quantity\;of\;sucrose \cr & \;solution\;consumed \cr} \over \eqalign{& The\;quantity\;of\;sucrose\;solution\;consumed \cr & + The\;quantity\;of\;water\;consumed\; \cr} } \cr} $$

### Tail suspension test (TST)

The TST was performed following a method outlined in prior work by Steru [26]. In this procedure, each mouse was suspended by its tail approximately 75 cm above the floor, using adhesive tape placed about 1 cm from the tip of the tail. The total immobility duration was recorded for 6 min. The mice were regarded as immobile only when they either hung passively or remained completely motionless [26].

### Forced swimming test ced swimming test (FST)

The FST was performed following the protocol previously established in our laboratory [20]. Mice were forced to swim individually in glass cylinders (25 cm in height and 10 cm in diameter) filled with water (10 cm deep) at 23 °C. The animals remained in the cylinder for 6 min. The total duration of immobility was recorded during the last 4 min of the test. Immobility was recognized when a mouse floated passively in an upright position with its head positioned above the water level [27].

### Locomotor activity test

The spontaneous locomotor activity was recorded individually for each animal according to the previously described procedure to eliminate the non-specific effects of scopolamine at its effective dose [28]. The measurement system consisted of plexiglas locomotor activity cages (13 × 23 × 15 cm) in a photobeam activity system (Opto-M3 Activity Meter, Columbus Instruments, USA), where the animals were individually placed 45 min after drug injection. After each trial, the cages were thoroughly washed and dried. The total number of ambulations was recorded in 5-minute intervals fora total of 30 min. Each group consisted of 8 mice.

### Synaptosome fraction preparation

Tissue samples from the PFC and hippocampus were homogenized using ice-cold lysis buffer composed of 0.32 M sucrose, 20 mM HEPES (pH 7.4), 1× protease inhibitor cocktail, 5 mM NaF, 1 mM NaVO3, and 1 mM EDTA. Homogenates were centrifuged at 2800 rpm for 10 min at 4 °C. The obtained supernatant was then centrifuged at 12,000 rpm for 10 min at 4 °C. Pellets obtained from this second centrifugation were then sonicated in a protein lysis buffer containing 50 mM Tris-HCl (pH 7.5), 150 mM NaCl, 1% Triton X-100, 0.1% SDS, 2 mM EDTA, 1 mM NaVO3, 5 mM NaF, and a protease inhibitor cocktail. The protein concentrations were quantified using a commercially available BCA kit (Thermo Scientific, USA).

### Western blotting

Thirty micrograms of protein from each sample were separated by SDS-polyacrylamide gel electrophoresis (10%) and then transferred onto nitrocellulose membranes (Millipore, Bedford, MA, USA). These membranes were blocked for 1 h using a 1% blocking solution (BM Chemiluminescence Western Blotting Kit (Mouse/Rabbit), cat. no. 11,520,709,001, made by Roche, Switzerland). Following the blocking step, the membranes were incubated overnight at 4 °C with the primary antibodies. The primary antibodies used included anti-mTOR (mTOR 1:1000; cat. no. 2972 S, Cell Signaling Technology, USA), anti-phospho-mTOR (pmTOR, S2481, 1:1000; ab137133, Abcam, USA), anti-phospho-eEF2 (pheEF2 (phospho T56) 1:1000; ab53114, Abcam, USA), anti-eEF2 (eEF2 1:1000; ab33523, Abcam, USA), anti-PSD95 (PSD 1:1000; ab12093, Abcam, USA), anti-TrkB (TrkB 1:1000; 4603 S, Cell Signaling Technology, USA). On the following day, the membranes were washed three times for 10 min in Tris-buffered saline with Tween (TBS-T) and incubated for 60 min with corresponding secondary IgG-peroxidase-conjugated antibodies: horse anti-mouse IgG (1:1000; PI-2000, Vector Laboratories, USA), goat anti-rabbit IgG (1:1000; PI-1000, Vector Laboratories, USA), rabbit anti-goat IgG (1:5000; ab6741, Abcam, USA). After this incubation, the membranes were washed three times for 10 min with TBS-T. In the final step, the blots were incubated with a detection reagent (Bio-Rad, USA). The signal emanating from the tested proteins was captured and quantified using a Fuji Las 1000 system and Fuji Image Gauge V4.0 software. A primary monoclonal antibody, specifically glyceraldehyde 3-phosphate dehydrogenase (GAPDH, 1:500; MAB374, Millipore, Germany), was indicated on each blot to verify the transfer and loading accuracy. The final result is presented as the ratio of the optical density of a particular protein to the optical density of GAPDH. Each experiment was replicated twice for reliability.

### Statistical analysis

Statistical analyses were performed using GraphPad Prism 9.1.2 (GraphPad Software, San Diego, CA, USA). All data are expressed as the mean ± standard error of the mean (SEM). Number of mice used varied across groups between 8 and 10. The distribution of variables and the homogeneity of variances were checked by the Shapiro–Wilk’s test and Levene’s test, respectively. Two-way ANOVA followed by Bonferroni’s post hoc test was used to analyze the interactions between UCMS and scopolamine in each behavioral test. Data illustrating locomotor activity were evaluated by repeated measures ANOVA followed by Bonferroni’s multiple comparisons test. Two-way ANOVA followed by Bonferroni’s post hoc test was used to analyze Western blotting data. P values lower than 0.05 were regarded as statistically significant.

## Results

### Behavioral effects of single administration of scopolamine in the UCMS model of depression

**Fig. 2:**
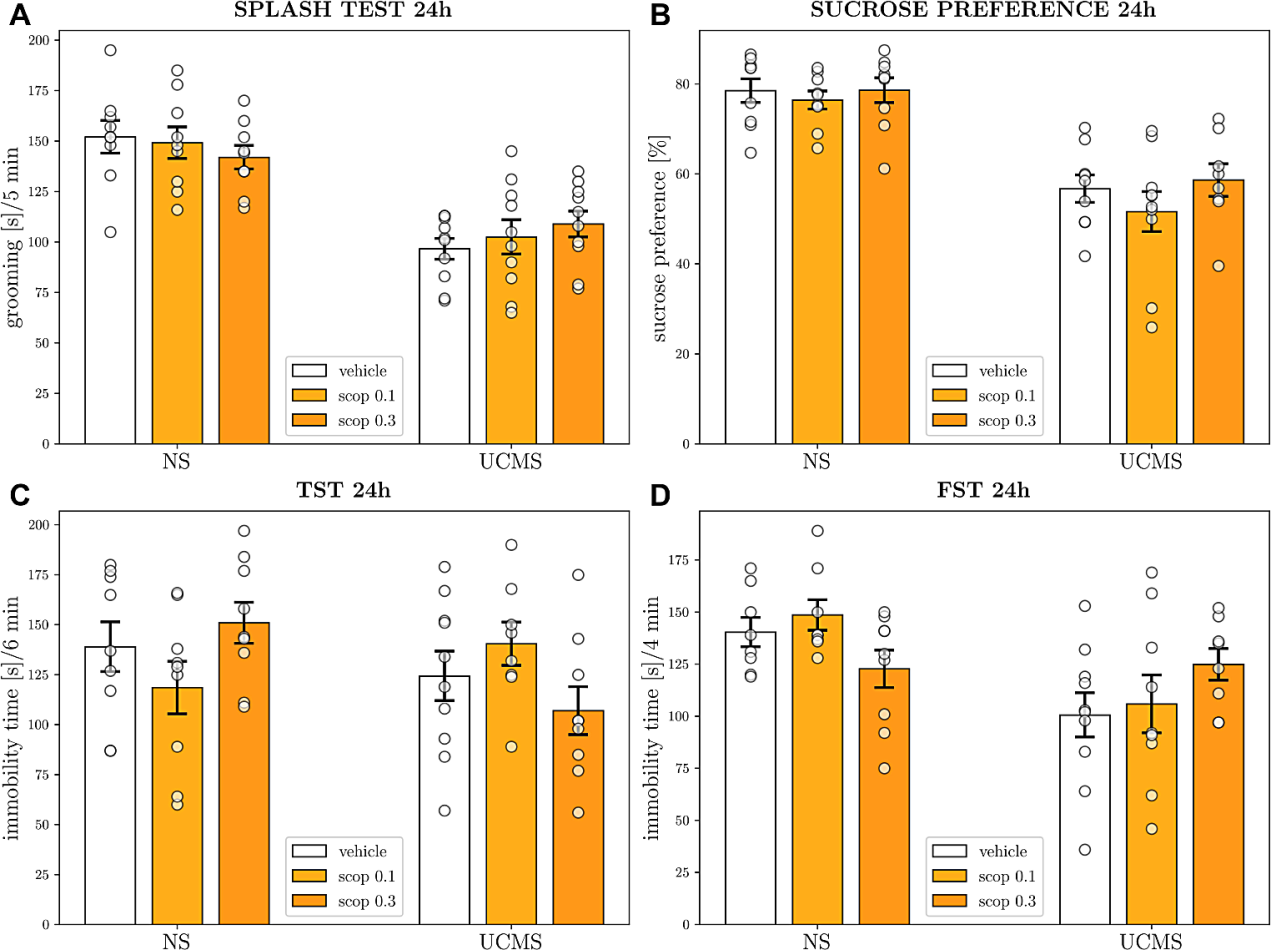
The antidepressant-like effects of a single administration of scopolamine in the UCMS model of depression. Behavioral effects in **(A)** splash test; **(B)** sucrose preference test (SPT); **(C)** tail suspension test (TST); and (D) forced swim test. The values are expressed as the means ± SEM (*N* = 8–10) and were analyzed by two-way ANOVA followed by Bonferroni’s multiple comparisons test. NS– non-stressed; UCMS– unpredictable chronic mild stress

In the splash test, a two-way ANOVA showed no interaction between UCMS procedure and scopolamine treatment [*F*_2,51_ = 1.275, *p* = 0.2881; Fig. 2A]. In the SPT, no interaction between UCMS and scopolamine was also observed [*F*_2,48_ = 0.2875, *p* = 0.7514; Fig. 2B]. In the TST, a two-way ANOVA did not reveal an interaction between UCMS and scopolamine [*F*_2,47_ = 3.054, *p* = 0.0566; Fig. 2C]. In the FST, a two-way ANOVA showed no interaction between UCMS procedure and scopolamine treatment as well [*F*_2,46_ = 3.170, *p* = 0.0513; Fig. 2D].

### Behavioral effects of four-day administration of scopolamine in the UCMS model of depression

**Fig. 3:**
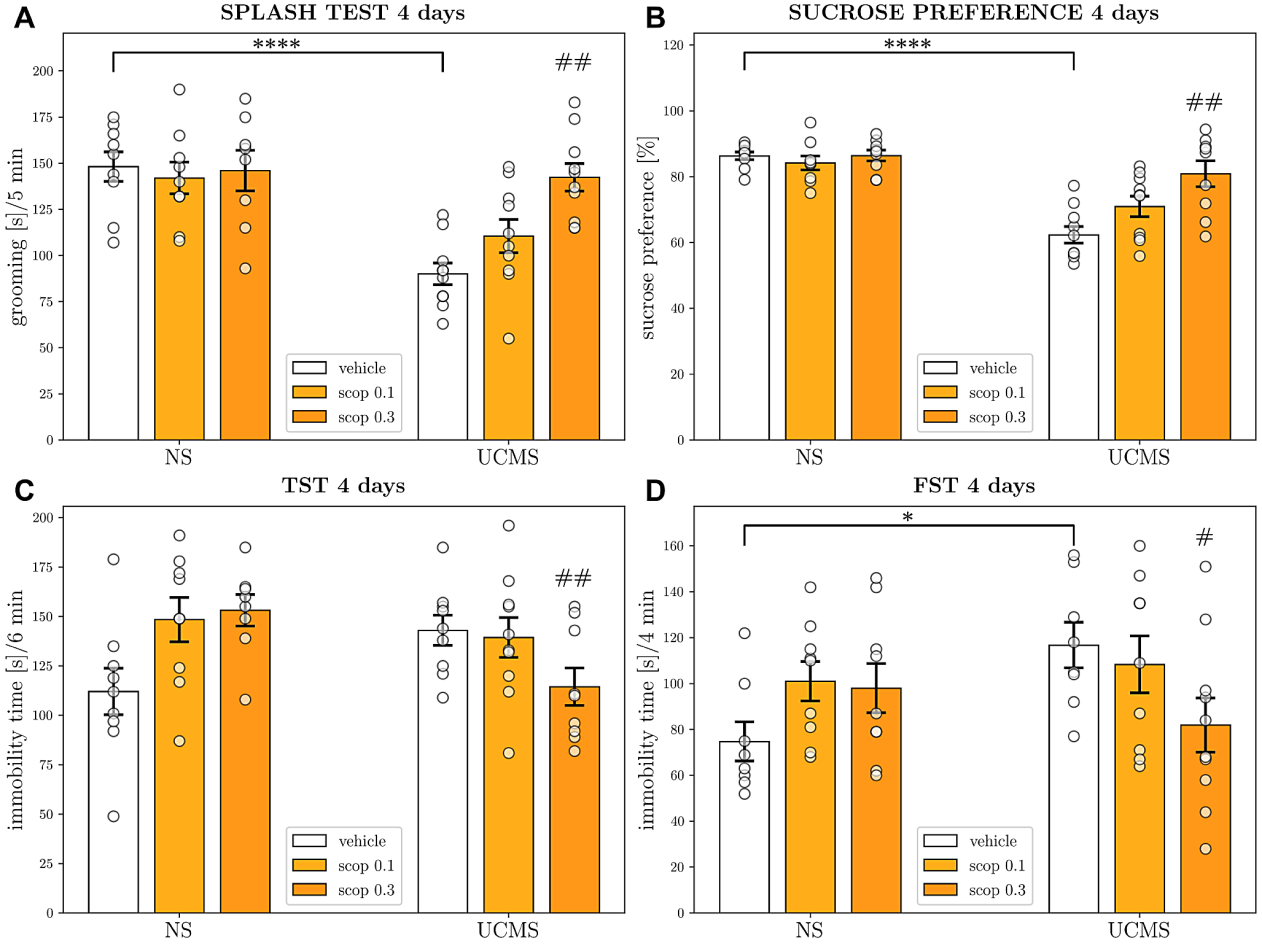
The antidepressant-like effects of four-day administration of scopolamine in the UCMS model of depression. Behavioral effects in **(A)** splash test; **(B)** sucrose preference test (SPT); **(C)** tail suspension test (TST); and **(D)** forced swim test. The values are expressed as the means ± SEM (*N* = 8–10) and were analyzed by two-way ANOVA followed by Bonferroni’s multiple comparisons test. ∗*p <* 0.05; ∗∗∗∗*p <* 0.0001 vs. the respective NS vehicle; #*p <* 0.05; ##*p <* 0.01 interaction: UCMS × scop. NS– non-stressed; UCMS– unpredictable chronic mild stress

The influence of a four-day scopolamine injection (0.1 and 0.3 mg/kg) on the behavioral effects induced by UCMS was also investigated. In the splash test, two-way ANOVA revealed an interaction between UCMS and scopolamine [*F*_2,50_ = 5.288, *p* = 0.0083; Fig. 3A], and effect of both UCMS procedure [*F*_1,50_ = 20.84, *p* < 0.0001], and scopolamine treatment [*F*_2,50_ = 4.741, *p* = 0.0130], indicating a reversal of the UCMS-induced apathy-like state. Bonferroni post-hoc test showed a significant shortening of the grooming time in the mice subjected to the UCMS procedure compared to the NS controls (*p* < 0.0001). Similar results were found in the SPT, where statistical analysis revealed a notable interaction between UCMS and scopolamine [*F*_2,50_ = 6.236, *p* = 0.0038; Fig. 3B], and effect of both UCMS procedure [*F*_1,50_ = 44.50, *p* < 0.0001], and scopolamine treatment [*F*_2,50_ = 6.497, *p* = 0.0031], suggesting a reversal of the UCMS-induced anhedonia-like state after four-day scopolamine administration. Bonferroni post-hoc test indicated a significantly decreased sucrose preference in stressed mice (*p* < 0.0001). In the TST, two-way ANOVA showed an interaction between UCMS and scopolamine [*F*_2,48_ = 6.048, *p* = 0.0045; Fig. 3C], but no effect of scopolamine [*F*_2,48_ = 1.431, *p* = 0.2490] or stress [*F*_1,48_ = 0.4797, *p* = 0.4919] was observed. Bonferroni post-hoc test did not reveal changes in immobility time of stressed mice compared to that of the unstressed controls (*p* = 0.0967). In the FST, two-way ANOVA showed an interaction between UCMS and scopolamine [*F*_2,47_ = 3.683, *p* = 0.0327; Fig. 3D], and Bonferroni post-hoc test revealed a significantly increased immobility time of stressed mice compared to that of the unstressed controls (*p* = 0.0320). Although no effect of UCMS [*F*_1,47_ = 1.620, *p* = 0.2094, or scopolamine [*F*_2,47_ = 1.012, *p* = 0.3712] was observed.

### The effects of acute scopolamine treatment on the locomotor activity of non-stressed mice

**Fig. 4:**
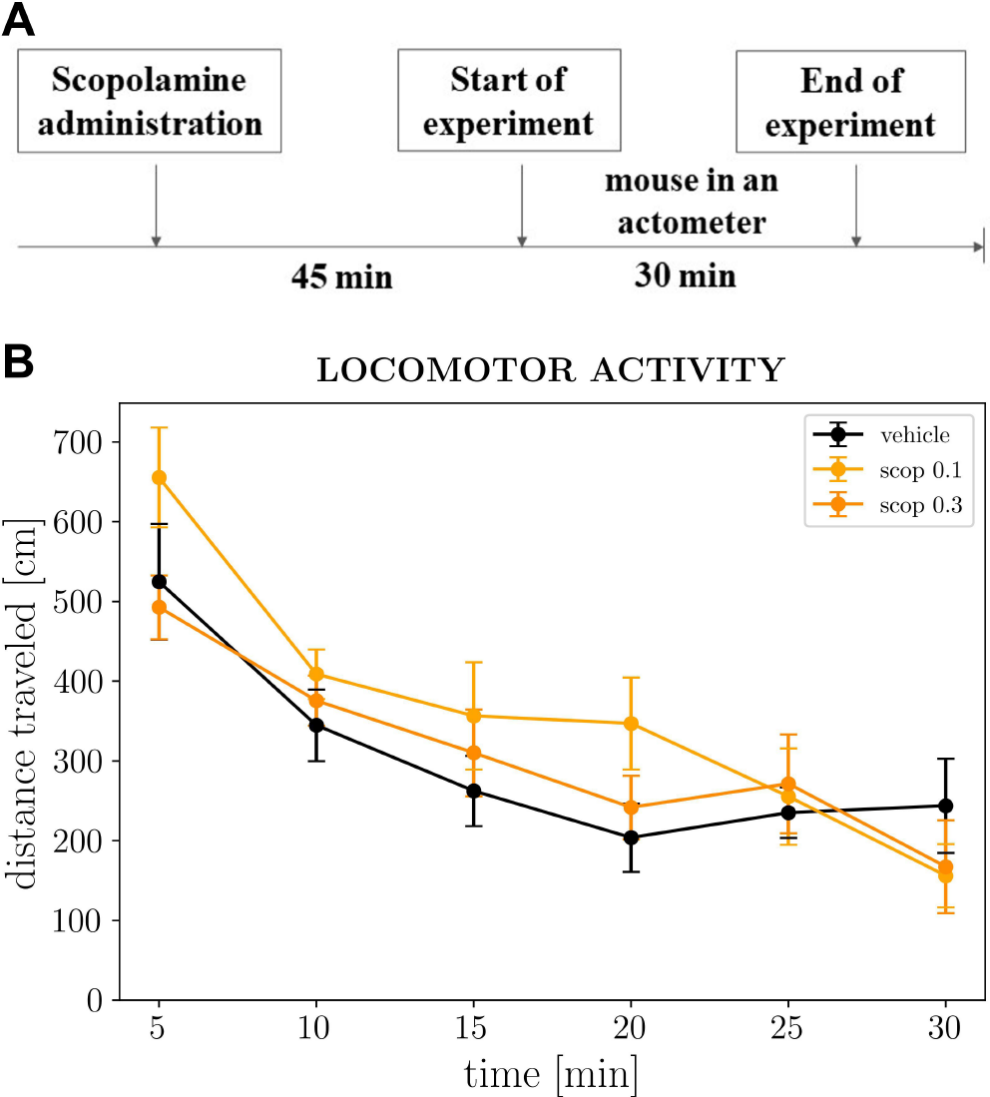
The effect of scopolamine on the locomotor activity in C57BL/6J mice. **(A)** Schematic representation of the experimental schedule. **(B)** The effect of a single administration of scopolamine on the spontaneous locomotor activity of C57BL/6J mice during a 30-min experimental session. The values are expressed as the means ± SEM (*N* = 8) and were analyzed by repeated measures ANOVA

To eliminate any non-specific effects of the antidepressant drugs that could interfere with the results of behavioral tests based on immobility time following initial escape-oriented movements (such as TST and FST), the locomotor activity measurement was performed on non-stressed mice treated acutely with either scopolamine or a vehicle 45 min prior to the test. Two-way repeated measures ANOVA showed that scopolamine at doses of 0.1 and 0.3 mg/kg did not reveal any impact on the distance traveled ([*F*_1,14_ = 1.133, *p =* 0.3052] and [*F*_1,14_ = 0.01809, *p =* 0.8949], respectively) compared to control (Fig. 4B). However, the locomotor activity of mice was time-dependent, gradually decreasing over the course of the 30-minute experimental session. Two-way repeated measures ANOVA showed a main time effect [*F*_3,47_ = 41.26, *p* < 0.0001] for mice that received the 0.1 mg/kg dose and [*F*_4,51_ = 31.67, *p* < 0.0001] for mice that received the 0.3 mg/kg dose (Fig. 4B).

### The effects of scopolamine four-day administration on the level of proteins involved in synaptic plasticity processes in the PFC

**Fig. 5:**
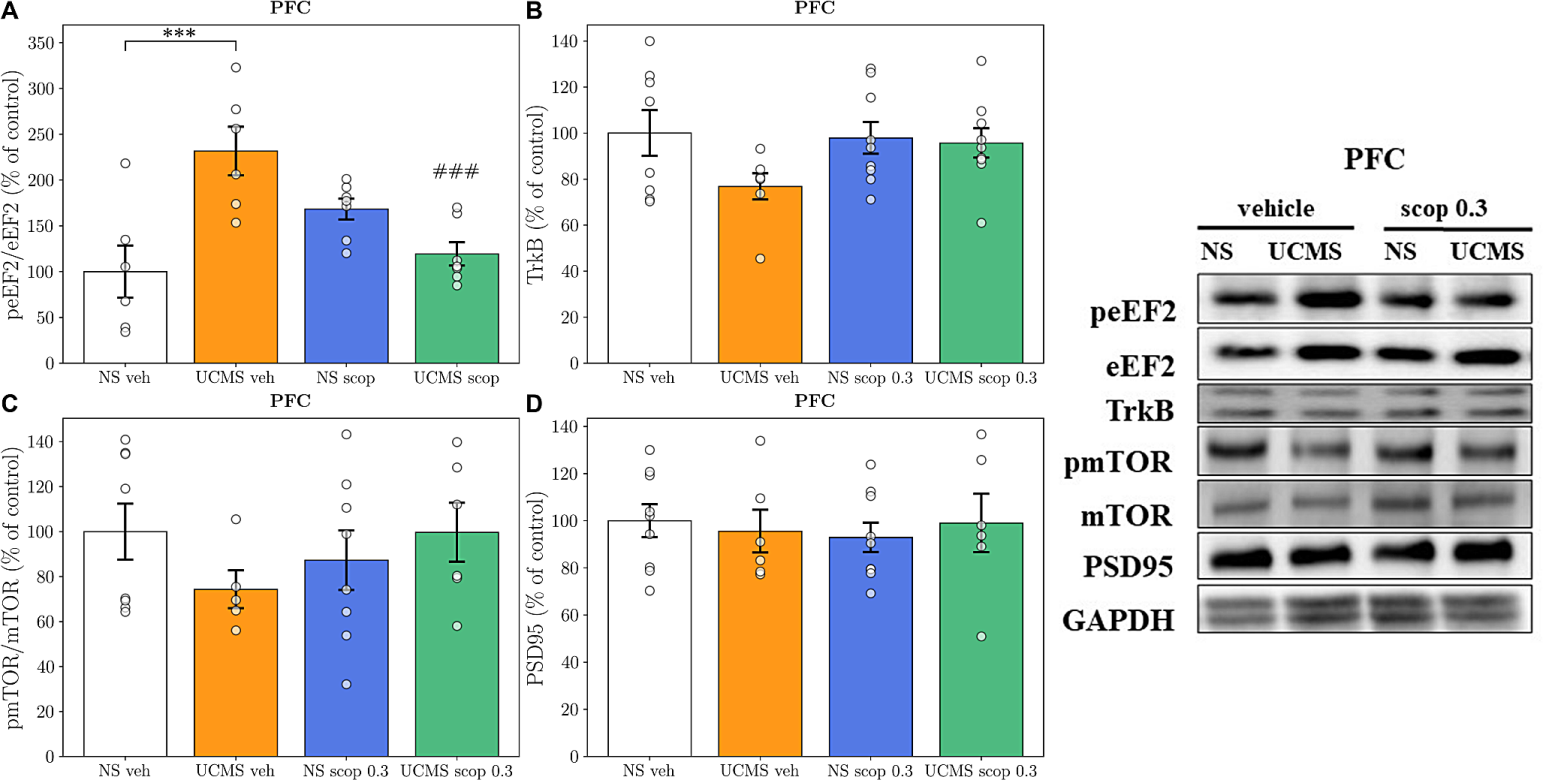
The effect of a four-day administration of scopolamine (0.3 mg/kg) on the **(A)** peEF2/eEF2, **(B)** TrkB, **(C)** pmTOR/mTOR, and **(D)** PSD95 protein levels analyzed by Western blot analysis in the synaptosome-enriched fraction of the PFC. The values are expressed as percentage of changes vs. control levels (*N* = 6–9) and were analyzed by two-way ANOVA followed by Bonferroni’s post hoc test. ∗∗∗*p <* 0.001 vs. the respective NS vehicle; ###*p <* 0.001 interaction: UCMS × scop. NS– non-stressed; UCMS– unpredictable chronic mild stress

In the PFC, a two-way ANOVA revealed an interaction between UCMS and scopolamine at a dose of 0.3 mg/kg [*F*_1,22_ = 20.13, *p* = 0.0002], indicating a reversal of the UCMS-induced changes in phospho-eEF2/eEF2 ratio. Bonferroni post-hoc test indicated a significantly increased phospho-eEF2/eEF2 ratio in stressed mice compared to the NS controls (*p* = 0.0004). Statistical analyses of Western blots in the PFC did not reveal any interaction between UCMS and scopolamine regarding the level of TrkB, mTOR, and PSD95 proteins ([*F*_1,29_ = 1.974, *p* = 0.1706]; [*F*_1,23_ = 2.158, *p* = 0.1554], and [*F*_1,27_ = 0.4304, *p* = 0.5173] for Fig. 5B and D, respectively). However, a clear trend to decrease the TrkB level between the vehicle group and UCMS group was observed (*p* = 0.0894, for Fig. 5B).

### The effects of scopolamine four-day administration on the level of proteins involved in synaptic plasticity processes in the hippocampus

**Fig. 6:**
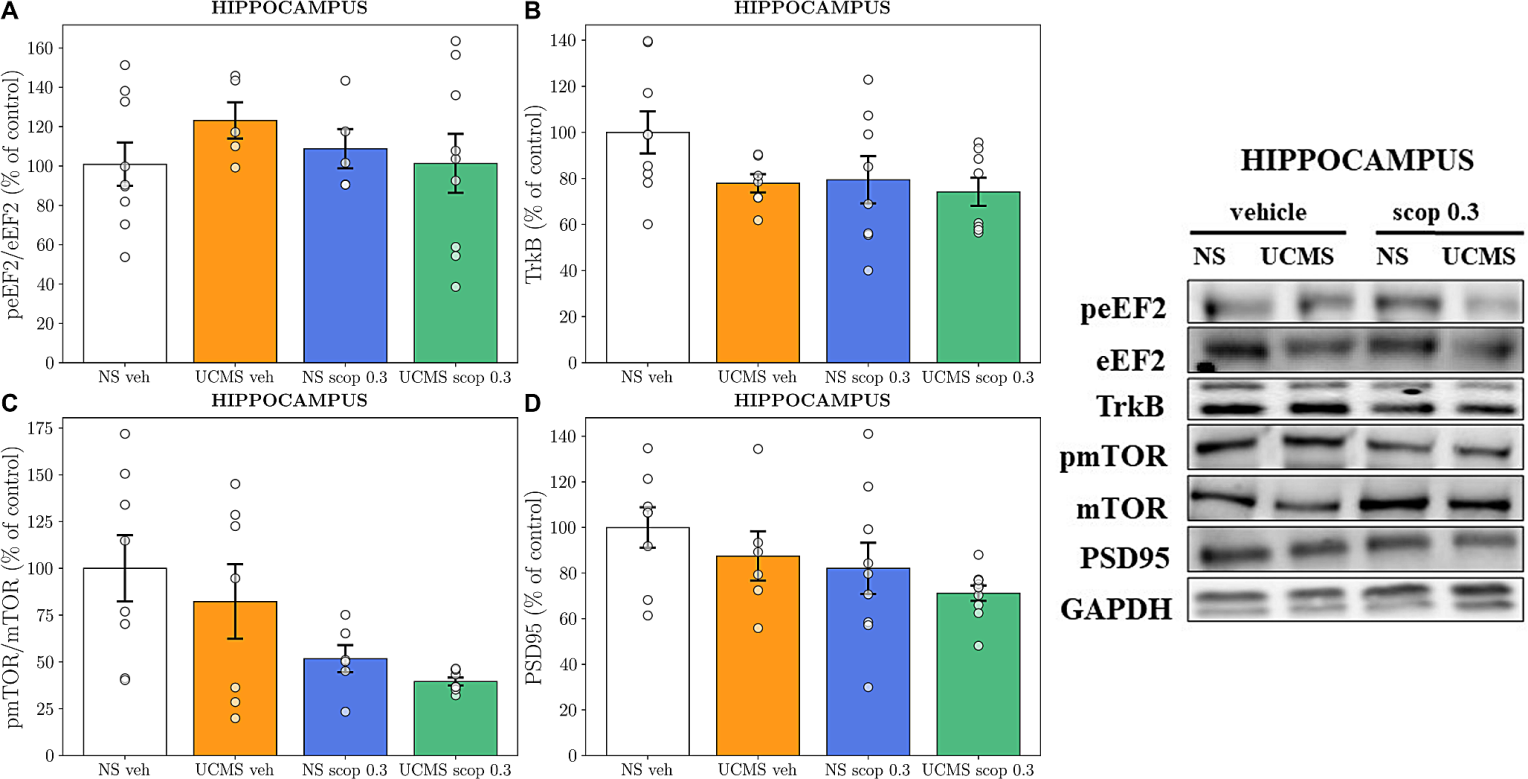
The effect of a four-day administration of scopolamine (0.3 mg/kg) on the **(A)** peEF2/eEF2, **(B)** TrkB, **(C)** pmTOR/mTOR, and **(D)** PSD95 protein levels analyzed by Western blot analysis in the synaptosome-enriched fraction of the hippocampus. The values are expressed as percentage of changes vs. control levels (*N* = 6–9) and were analyzed by two-way ANOVA followed by Bonferroni’s post hoc test. NS– non-stressed, UCMS– unpredictable chronic mild stress

In the hippocampus, two-way ANOVA did not show any interaction between UCMS and scopolamine ([*F*_1,24_ = 1.190, *p* = 0.2861]; [*F*_1,28_ = 1.086, *p* = 0.3062]; [*F*_1,24_ = 0.03441, *p* = 0.8544]; [*F*_1,29_ = 0.008, *p* = 0.9283] for Fig. 6A and D, respectively), suggesting that level of tested proteins was unaffected by above factors.

## Discussion

The present research shows that scopolamine induces antidepressant-like effects in a dose-dependent manner only after subchronic, but not single, administration in the UCMS model of depression in C57BL/6J mice, without affecting locomotor activity. Scopolamine treatment was accompanied by eEF2 protein dephosphorylation and its subsequent reactivation in the PFC.

The antidepressant-like activity of scopolamine has been investigated in previous studies using screening tests [20, 29, 30], as well as various animal models of depression [31, 32]. Rodent models of depression are a valuable tool for testing potential new targets with antidepressant properties and investigating their mechanism of action. To normalize the effects of scopolamine treatment and study its mechanism of action, as well as to extend these investigations to other anticholinergic agents in the future, we chose the unpredictable chronic mild stress (UCMS) model of depression. The UCMS model allows us not only to measure parameters reflecting core symptoms of depression, such as apathy, anhedonia, or behavioral despair, but also to accurately predict the time required for a test compound to produce therapeutic effects in humans. This feature facilitates the differentiation between classical and rapid-acting antidepressants tested. Nevertheless, in establishing an experimental scheme and aiming for high reproducibility in the UCMS model, several variables must be considered, including the type and duration of stressors and the strain of mice used. C57BL/6J mice are the preferred choice due to their high susceptibility to UCMS and responsiveness to antidepressant treatment. However, it is important to note that the C57BL/6J strain may not be recommended for studies requiring an extended UCMS procedure, as it can develop adaptive changes to long-term UCMS procedures [33].

Depression-like behavior was assessed through the splash test, SPT, TST, and FST. Our data indicate that utilized UCMS protocol effectively induced depressive-like behavior, as evidenced by reduced grooming time in the splash test reflecting apathy, decreased sucrose preference indicative of anhedonia, and increased immobility time in the TST and the FST resembling behavioral despair. Interestingly, differential modulation of behaviors was observed in UCMS mice treated once versus subchronically with a vehicle. Namely, stressed mice treated with a vehicle for four days showed an expected increase in immobility time in both TST and FST tests. In contrast, mice treated with vehicle only once showed a decrease in immobility time. These effects may be due to the less persistent escape-oriented behaviors evoked by the stress protocol compared to anhedonia-based behavior or apathy-like states. Additionally, subchronic drug administration as an additional stressor could reduce the risk of adaptations to stress, maintaining the effects of the UCMS protocol even a few days after the last day of the stress procedure, when TST and FST tests were performed.

Subchronic administration of scopolamine was found to be the necessary minimum required to alleviate these symptoms and induce rapid antidepressant effects. Specifically, scopolamine administered at a dose of 0.3 mg/kg for four consecutive days significantly reversed the UCMS-induced behaviors. In contrast, a single administration of scopolamine at the same dose did not alleviate the previously mentioned depressive-like symptoms. Many studies on animals demonstrate antidepressant-like effects just after a single administration, whereas our results show that such dosing is not adequate and should be changed to subchronic dosing. This pattern aligns with clinical data describing the relatively rapid (within three days) efficacy of scopolamine in patients with major depressive disorder (MDD) or bipolar disorder [12, 15]. Moreover, the observed antidepressant-like effects persisted for at least three days after the last treatment, suggesting potential prolonged effects of scopolamine treatment.

Importantly, subchronic administration of scopolamine effectively reverses UCMS-induced depressive-like behavior without influencing the behavior of the non-stressed group. This mirrors the observed efficacy of scopolamine in depressed patients and its lack of effect on healthy individuals. The connection between acetylcholine and the pathogenesis of depression is well-established [34]. Acetylcholine serves as the tie-link linking the sympathetic nervous system responsible for the ‘fight or flight’ response and the parasympathetic nervous system responsible for the ‘rest and digest’ processes. Stress, a key trigger for depression, disrupts the balance between these states. Clinical studies have reported elevated acetylcholine levels in individuals suffering from depression [34]. On the other hand, studies on unstressed rats have shown significant increases in acetylcholine release in both the frontal cortex and hippocampus [35]. It suggests that scopolamine’s capability to reverse depressive-like behavior could be specific only to stressed animals due to an upregulated cholinergic system.

Furthermore, we investigated the antidepressant-like mechanism of scopolamine by examining TrkB, mTOR, eEF2, and PSD95 levels in the PFC and hippocampus through Western Blot analysis. Notably, changes in the eEF2 protein level were observed in the PFC. Specifically, chronic stress increased eEF2 phosphorylation at Thr56, thereby reducing the translation elongation efficiency [36] and altering protein synthesis and processes associated with synaptic plasticity. In contrast, subchronic administration of scopolamine significantly decreased eEF2 phosphorylation, leading to its reactivation. Interestingly, such effects were not observed in the hippocampus, showing only a change trend. This suggests that eEF2 dephosphorylation in the PFC may play a crucial role in the mechanism underlying the prolonged antidepressant-like effect of scopolamine. These changes might be linked to the process known as systems memory consolidation, as the translation elongation factor eEF2 is known to be involved in hippocampus-dependent cognitive functions [37]. After learning, for example, in response to signals arising from exposure to a stressor, the memory engram is primarily encoded in the hippocampus but subsequently stabilized in other brain regions, such as the cortex, for long-lasting storage. Meanwhile, the memory engram in the hippocampus is likely to wane [38]. Synaptic plasticity is the fundamental mechanism underlying learning and memory, which may correlate with the level of eEF2 phosphorylation, depending on the duration of exposure to stress.

Interestingly, the remaining Western blot analyses revealed no differences in TrkB, PSD95, and phosphorylated mTOR levels in both the hippocampus and PFC. However, a robust non-statistical tendency of scopolamine treatment to restore the level of TrkB protein, which was decreased by chronic stress, was observed in PFC. This observation aligns with literature reports and supports the hypothesis regarding the role of TrkB receptor in the mechanism of action of rapid-acting antidepressants [39–42]. Given the importance of BDNF-TrkB signalling in mediating physiological functions, including cell survival and synaptic plasticity, alterations in BDNF-TrkB signaling could be implicated in the pathophysiology of mood disorders in humans [43, 44]. Postmortem studies have demonstrated a decreased expression of BDNF and TrkB in the prefrontal cortex and hippocampus of suicide subjects with major depression [45]. Conversely, antidepressant treatment increases brain and serum BDNF levels in depressed patients, which could subsequently initiate signaling via TrkB receptors [46, 47]. Recent studies suggest that the eukaryotic elongation factor 2 (eEF2) and the inhibition of its phosphorylation by its dedicated kinase, eukaryotic elongation factor 2 kinase (eEF2K), may play a pivotal role in the rapid antidepressant action of ketamine [48]. eEF2 is involved in translation elongation, and its dephosphorylation leads to the de-suppression of protein synthesis, particularly BDNF, and the subsequent initiation of synaptic plasticity processes [49]. This raises the question: could eEF2 be a universal signaling target of rapid-acting antidepressant drugs, responsible for triggering downstream signaling effects that elicit rapid and sustained homeostatic synaptic plasticity? Our findings suggest a role for eEF2 in the rapid antidepressant action of scopolamine. However, the limited biochemical findings prevent us from suggesting a clear mechanism of scopolamine’s effects on UCMS-induced depression at this time. Therefore, further research on BDNF-TrkB signaling is needed to better understand the underlying reasons for the observed effects.

## Conclusion

This study indicates that the administration of scopolamine is effective in alleviating UCMS-induced depressive-like behavior, but a subchronic, specifically four-day, administration is necessary for this effect. This antidepressant-like effect depends on eEF2 protein activity in the prefrontal cortex, a region implicated in synaptic activity processes. The research successfully establishes the suitability of the unpredictable chronic mild stress (UCMS) model of depression in normalizing the effects of scopolamine treatment. It opens the door to further investigations into the mechanisms of rapid-acting antidepressants.

## Electronic supplementary material

Below is the link to the electronic supplementary material.

Supplementary Material 1

## Data Availability

All relevant data are presented in the manuscript, raw data are available upon request from the corresponding author.

## Abbreviations

eEF2: Eukaryotic elongation factor 2
FST: Forced swimming test
mTOR: Mammalian target of rapamycin
PFC: Prefrontal cortex
PSD95: Postsynaptic density protein 95
SPT: Sucrose preference test
TrkB: Tropomyosin receptor kinase B
TST: Tail suspension test
UCMS: Unpredictable chronic mild stress
