# Supplementary material for: Subchronic administration of scopolamine reverses UCMS-induced behavior in mice via eEF2 protein dephosphorylation
Source: Pharmacol Rep. 2024 Jul 23;76(5):1001–11. doi: 10.1007/s43440-024-00630-4 (PMC11387448; doi:10.1007/s43440-024-00630-4)
Supplement: Supplementary file 1 — Supplementary Material 1 [file 43440_2024_630_MOESM1_ESM.docx]

**Fig. S1**. The effect of a four-day administration of scopolamine (0.3 mg/kg; *ip*) on the eEF2, TrkB, mTOR, and PSD95 protein levels determined by Western blot analysis in the synaptosome-enriched fraction of the prefrontal cortex (PFC). Raw, unprocessed Western blot images. GAPDH – reference protein.

**
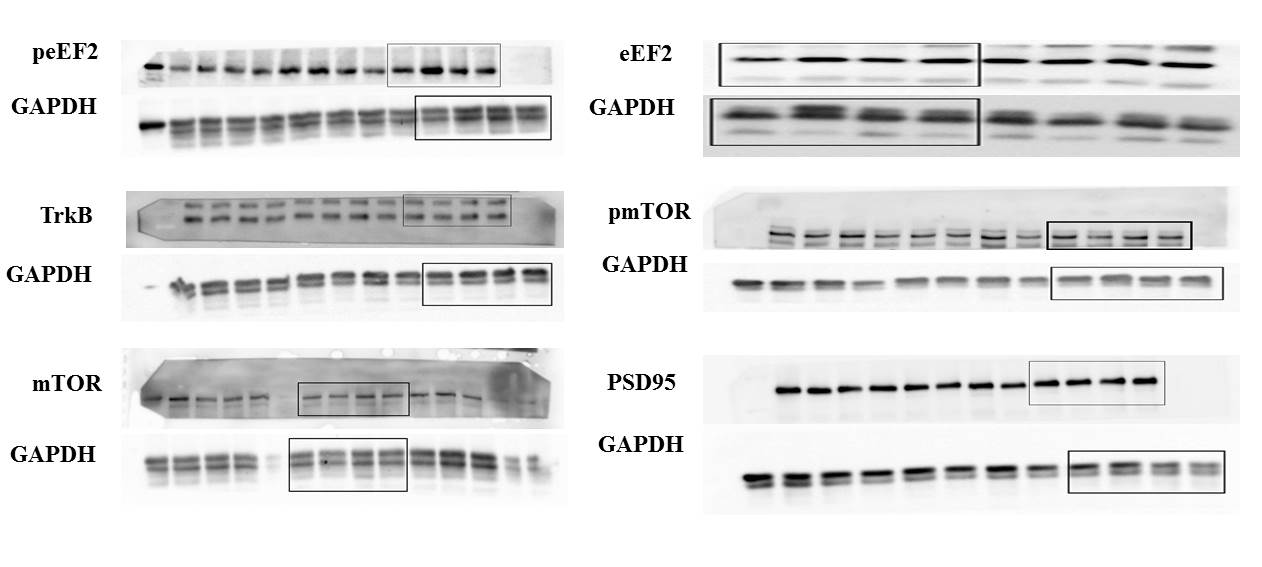
**

**Fig. S2**. The effect of a four-day administration of scopolamine (0.3 mg/kg; *ip*) on the eEF2, TrkB, mTOR, and PSD95 protein levels determined by Western blot analysis in the synaptosome-enriched fraction of the hippocampus. Raw, unprocessed Western blot images. GAPDH – reference protein.

**
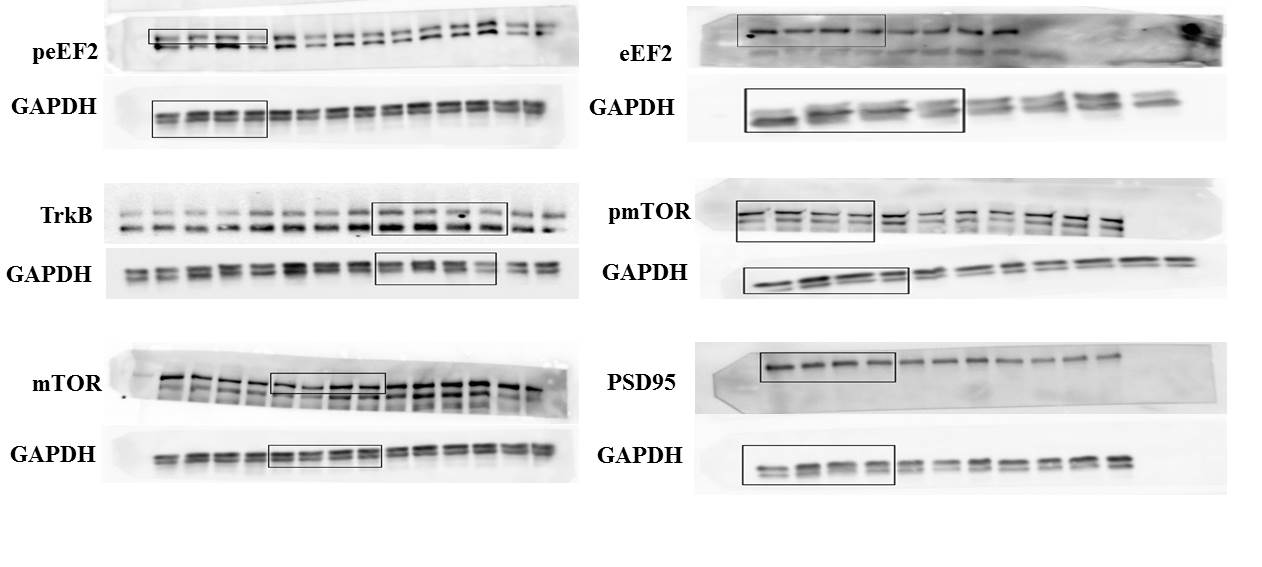
**
